# *Dolphin Morbillivirus*: a lethal but valuable infection model

**DOI:** 10.1038/emi.2013.74

**Published:** 2013-11-06

**Authors:** Giovanni Di Guardo, Sandro Mazzariol

**Affiliations:** 1 University of Teramo, Faculty of Veterinary Medicine, Piazza Aldo Moro, 45-64100 Teramo, Italy; 2 University of Padua, Department of Comparative Biomedicine and Food Science, Agripolis, Viale dell'Università, 16-35020 Legnaro (PD), Italy

## Abstract

*Dolphin Morbillivirus* (DMV), which has caused at least four epidemics in the Western Mediterranean during the last 20–25 years, may dramatically impact the health and conservation of striped dolphins (*Stenella coeruleoalba*) living in this area. The viral and host factors driving the host–DMV interaction, along with those related to the climate change that underlie the occurrence of DMV epidemics, warrant further investigation.

From 1990 onward, Mediterranean striped dolphins (*Stenella coeruleoalba*) have been the victims of at least four *Dolphin Morbillivirus* (DMV) epidemics, and the epidemic that occurred between 1990 and 1992 was particularly dramatic. Noteworthy, several infected animals showed the presence of viral genomes and/or antigens exclusively in the brain, and their lesions mimicked those found in *Measles Virus* (MeV)-infected, subacute sclerosing panencephalitis-affected patients.^1,2^ Furthermore, an expansion in the host-range of this virus has been recently observed in the Western Mediterranean, as highlighted by lethal cases of DMV infection in two fin whales (*Balaenoptera physalus*) that were stranded along the Tyrrhenian coast of Italy^1^ and in a captive, common seal (*Phoca vitulina*) from an Italian aquatic park.^3^ These findings are of concern, given the documented susceptibility of Mediterranean monk seals (*Monachus monachus*), which is a critically endangered species, to an agent that is closely related to DMV.^4^

The viral strains that were responsible for these epidemics, including the dramatic outbreak that occurred in the striped dolphin population of the Tyrrhenian Sea from January to April 2013, exhibited a marked genetic relatedness.^1,3^ This finding argues in favor of prolonged DMV circulation in the Western Mediterranean and simultaneously supports the hypothesis stating that an inadequate level of anti-viral immunity exists in the striped dolphins that inhabit this area. Based on the available data, it can be predicted that cyclic DMV epidemics should be occurring every 3–5 years in the Western Mediterranean. A similar scenario, which is made plausible by the number of young individuals supporting the spread of infection and by unsatisfactory population immunity against the virus, could tremendously impact the already endangered health and conservation status of Mediterranean striped dolphins. In this respect, it is unknown whether the high tissue levels of immunotoxic pollutants that are commonly detected in Mediterranean striped dolphins^5^ are responsible for the lack of protective immunity against one or more of the closely related DMV strains that have been putatively circulating for years in this region.

Additionally, the viral- and host-specific factors driving the host–DMV interaction are largely undefined, and special emphasis should be placed on the immunopathogenesis of infection and on the mechanisms of viral colonization and prolonged persistence inside the brain of chronically infected dolphins.^1^ Similar to all other *Morbillivirus* genus members, DMV is a lymphotropic, epitheliotropic and neurotropic agent. However, the cell receptor that accounts for morbilliviral lymphotropism (Signalling Lymphocyte Activation Molecule, SLAM, or CD150) is not expressed by neurons,^6^ and this may also be true for nectin-4, which is a cell receptor that is involved in *Morbillivirus* epitheliotropism;^6^ however, a role of such a molecule in the neurovirulence of *Canine Distemper Virus* (CDV) has been recently suggested.^7^ Indeed, CDV represents a well-established and ‘accredited' model for the comparative study of *Morbillivirus* infections in humans and animals, and, in fact, this agent gives rise to viral persistence inside the brain of infected dogs and, thereby, mirrors the behavior of MeV in human patients.^8^

Despite the above analysis, many questions remain unanswered. How does the virus colonize the brain of the host and its neuronal and non-neuronal cell populations? Additionally, how do morbilliviruses remain ‘undisturbed' inside the brain and give rise to MeV-induced subacute sclerosing panencephalitis^8^ in humans and its ‘disease analogues' in CDV-infected dogs^6,8^ and in DMV-infected striped dolphins?^1,2^ A plausible answer could lie in the ‘selective/exclusive neurotropic attitude' of given morbilliviral strains inside their hosts, and this phenomenon is likely determined by the selective interaction of the virus with a receptor molecule that is consistently expressed by neurons and/or by other brain cells. For example, during CDV infection, the prolonged persistence of the virus within the canine brain is associated with a viral, non-cytolytic, astrocyte-to-astrocyte intracellular spread via a hitherto unknown glial cell receptor.^9^ For human MeV infection, although a ‘nectin-4-based mechanism' could justify the neurotropism, neurovirulence and persistence of the virus inside the brain of infected patients,^10^ in a similar manner to what was reported in CDV-infected dogs,^7^ an alternative mechanism has been proposed. Albeit highly speculative, this ‘model' postulates an interaction between CD147, which is a transmembrane glycoprotein and a gamma-secretase subunit that is expressed by neuronal cells,^11^ and cyclophilin B, which is incorporated into the MeV surface.^6^

In regard to the agent-related factors that drive viral persistence inside the brain, it should also be emphasized that MeV, CDV and the more recently characterized *Phocine Distemper Virus* have been shown to undergo point- and hypermutation events in their viral envelope genes, such as those coding for fusion (F) and matrix (M) proteins.^8,12^ While it seems likely that the aforementioned gene mutations may contribute to prolonged morbilliviral persistence in the brain of chronically infected individuals, it is important to investigate whether a similar pathogenetic mechanism also occurs in DMV-infected dolphins.

A powerful research effort is needed that pays special attention to the fact that few studies thus far have investigated the entire viral genome of the isolates that were recovered from *Morbillivirus*-infected cetaceans. In addition to the lack of a financially and ethically sustainable experimental model for the study of DMV infection in dolphins, the research activity in this area, and in general, on the pathology of free-ranging cetaceans is hampered by the advanced degree of post mortem autolysis in which their bodies are often found in after stranding.

In conclusion, while recognizing the long-standing ‘tradition' and the outstanding value of the human MeV and dog CDV infection models, we believe that it is also important to characterize the virus- and host-specific factors that play a major role in the host–DMV interaction and the climate change-related drivers that influence the occurrence of cyclic DMV epidemics in the Mediterranean. This research topic is challenging and intriguing, and DMV-infected dolphins could potentially be valuable models for comparative neuropathology and viral neuropathogenesis.
